# The Combination of Mulberry Extracts and Silk Amino Acids Alleviated High Fat Diet-Induced Nonalcoholic Hepatic Steatosis by Improving Hepatic Insulin Signaling and Normalizing Gut Microbiome Dysbiosis in Rats

**DOI:** 10.1155/2019/8063121

**Published:** 2019-05-30

**Authors:** Sunmin Park, Ting Zhang, Jing Yi Qiu, Xuangao Wu

**Affiliations:** Department of Food & Nutrition, Obesity/Diabetes Center, Hoseo University, Asan, Republic of Korea

## Abstract

Mulberry water extracts (MB) and silk amino acids (SA) are reported to improve oxidative stress and inflammation, respectively. We hypothesized whether the mixture of mulberry water extracts and silk amino acids can alleviate nonalcoholic fatty liver disease (NAFLD) induced by high fat diets. Male Sprague Dawley rats were orally provided with high fat diets containing different ratios of MB and SA (1:3, MS1:3, or 1:5, MS1:5) or cellulose (the disease-control) for 12 weeks. Rats had 200 or 600 mg/kg bw of MS1:3 and MS1:5 (MS1:3-L, MS1:3-H; MS1:5-L, and MS1:5-H). Rats in the normal-control group were fed the 20% fat diet with cellulose. Disease-control rats exhibited much greater triglyceride (TG) deposition in the liver than the normal-control rats along with increased body weight gain, visceral fat mass, serum concentrations of cholesterol, triglyceride and nonesterified fatty acid (NEFA), and insulin resistance. Disease-control rats also had liver damage with increased oxidative stress and inflammation compared to the normal-control rats. MS1:3-H and MS1:5-H were found to have greater hepatic glycogen accumulation and decreased hepatic TG, insulin resistance, and dyslipidemia, with MS1:5-H being similar to the normal-control. MS1:3-H alleviated oxidative stress with lower hepatic lipid peroxide compared to MS1:5-H whereas MS1:5-H ameliorated inflammation and hepatocyte damage better than MS1:3-H. Both MS1:3-H and MS1:5-H potentiated hepatic insulin signaling (pAkt*⟶*pACC) and reduced the mRNA expression of TG synthesis genes mRNA (FAS and SREBP-1c). In the gut microbiome MS1:3-H elevated the ratio of* Bacteroidales* to* Clostridiales* in the cecum better than MS1:5-H but MS1:5-H reduced the proinflammatory* Turicibacterales*. In conclusion, both MS1:3-H and MS1:5-H prevented liver damage induced by high fat diets, mainly by suppressing oxidative stress and inflammation, respectively. MS1:3 and MS1:5 might be used as therapeutic agent for NAFLD.

## 1. Introduction

Nonalcoholic fatty liver disease (NAFLD) is a major liver disease worldwide [1]. Global prevalence of NAFLD is about 25.2% and it is highest in the Middle East and South America and lowest in Africa. The incidence of metabolic comorbidities associated with NAFLD includes 51.3% obesity, 22.5% type 2 diabetes, 69.2% dyslipidemia, 39.3% hypertension, and 42.5% metabolic syndrome. NAFLD includes simple hepatic steatosis, ≥5% of liver tissue steatotic [1].

NAFLD etiology is influenced by multiple factors such genetic, metabolic, and environmental factors [2]. Dietary factors are major contributors to the development of fatty liver, including ingestion of high fat and high fructose diets and toxic substances [3–5]. Recent research has demonstrated that intestinal dysbiosis also induces various metabolic diseases, and it can act as a trigger for NAFLD [3]. NAFLD is initiated by substrate overload, primarily sugar and fat, and consequent lipotoxic liver injury [5, 6]. The major trigger that initiates NAFLD is the consumption of excess dietary fats which results in the accumulation of nonesterified fatty acids that make potential lipotoxins [5].

Gut microbiota also modulate the absorption of monosaccharides in the intestinal cells, and the synthesis of short-chain fatty acids by microbiota digesting dietary fibers increases the secretion of peptide YY and glucagon-like-peptide-1, the hydrolysis of choline to dimethyl nitrosamine precursors, the suppression of bile acid production and, inflammation [7]. The gut microbiome response to excess sugar and lipids is associated with intestinal and systematic inflammation that leads to hepatic inflammation and induces hepatic steatosis when excess lipids are present [7, 8]. Thus, the modulation of gut microbiota may prevent the development of hepatic steatosis. NAFLD can be prevented or alleviated by stimulating energy utilization and by reducing inflammation and oxidative stress.

Mulberry (*Morus alba* L.) is well known to have antioxidant and anti-inflammatory activities. Our previous study has also shown that mulberry fruits protect against alcoholic liver steatosis, not only accelerating ethanol metabolism but also alleviating intestinal dysbiosis [9]. Silk amino acids have been reported to have antihyperglycemic and anti-inflammatory activities [10]. Both mulberry and silk amino acids may reduce oxidative stress and inflammation and alleviate damaged tissues additively or synergistically in our preliminary cell-based study. Therefore, different ratios of mulberry and mixtures of mulberry extract and silk amino acids may differently interact to alleviate nonalcoholic hepatic steatosis in rats. In the present study, we hypothesized that different mixtures of mulberry water extracts and silk amino acids may alleviate nonalcoholic hepatic steatosis in and rats. We tested the hypothesis in HepG2 cells treated with emulsified palmitate and rats with high fat diet-induced nonalcoholic hepatic steatosis and the action mechanism was explored.

## 2. Materials and Methods

### 2.1. Water Extract of Mulberry and Silk Amino Acids

Dried silkworm (*Bombys mori*) cocoon hydrolysates were obtained from Worldway Co. Ltd. (Sejong, Korea) and stored for further study. The silkworm cocoons were prepared by washing with 13-15 volume of water and hydrolyzing with 2N HCl at 100-110°C for 12h. The hydrolysates were filtered and salt contents in the hydrolysates were lowered to less than 0.3% at pH 5.5-7.5. The acid hydrolysates were sterilized and concentrated to 20-25 Brix in a low-pressure evaporator and then dried by a spray dryer. Amino acid contents were measured with an amino acid analyzer (L-8500; Hitachi, Tokyo, Japan) as described previously [11].

Dried Korean mulberry fruits (Worldway Co. Ltd, Sejong, Korea) were powdered and extracted in 5-fold volumes of water at 60°C for 2 h and the extracts were concentrated using a rotary evaporator. The concentrated extracts were lyophilized and the yield of mulberry extracts was 16.8%.

HPLC was performed using a JASCO liquid chromatography instrument (JASCO, Tokyo, Japan) equipped with an autoinjector, and a UV detector. The extract was analyzed with a YMC ODS-AM column (4.6 × 250 nm, 5 *μ*m, Waters). The mobile phase was 0.1% acetic acid in water (A) and 0.1% acetic acid in acetonitrile (B). The gradients were used as follows: 0 min, A:B 88:12 (v/v); 18 min, A:B 78:22; 28 min, A:B 72:28; 35 min, A:B 62:38; 48 min, A:B 52:48; 54 min, A:B 32:68; 58 min, 0:100. The mobile phase flow rate was 1.0 mL/min under the following conditions: column temperature, 35°C; injection volume, 20 *μ*L; and UV detection at 285 nm. The indicator compounds were cyanidin-3-glucoside, hydroxybenzoic acid, and rutin. Each indicator compound was purchased from Sigma (St. Louise, MO, USA) or ChromaDex (USA).

### 2.2. Effects of HepG2 Cell Line on Cell Damage

Human hepatocellular carcinoma HepG2 cells were acquired from American Type Culture Collection (HB-8065; Manassas, VA, USA) and were cultivated with high-glucose Dulbecco's modified Eagle's medium (DMEM) containing 10% fetal bovine serum (FBS) [12]. Cells were then transferred into 96-well plates at 4 × 10^3^ cells/well in high-glucose DMEM containing 0.3% bovine serum albumin (BSA) and allowed to grow to 70% confluence. The cells were administered with vehicle, 10 or 50 *μ*g/mL mulberry extracts (MB), silk amino acids (SA), or their mixtures as the ratio of MB and SA = 1:2 (MS1:2), 1:3 (MS1:3), and 1:5 (MS1:5). After 1 h of treatment, 0.5 mM palmitate emulsified with fatty acid free albumin was added to the HepG2 cells and they were incubated for another 24 h. The cell viability was determined by 3-(4,5-dimethylthiazol-2-yl)-2,5-diphenyl tetrazolium bromide (MTT) assay using an Aureon plate reader (Aureon Biosystems, Vienna, Austria).

HepG2 cells were grown in 24-well plates at 6 × 10^4^ cells/well until the cells were 70% confluent and then treated with vehicle, 10 or 50 *μ*g mL^–1^ MB, SA or their mixtures (MS1:2, MS1:3, and MS1:5) for 1 h. The cells were subsequently added with 0.5 mM palmitate and incubated for additional 24 h and the cells were lysed with lysis buffer at 4°C. The lysates were centrifuged at 10,000 x g for 20 min at 4°C and the supernatants were collected. Lipid peroxide levels as malondialdehyde (MDA) contents in the liver were then measured using a thiobarbituric acid reactive substances (TBARS) assay kit (Cayman Chemical, Ann Arbor, MI, USA). Relative mRNA levels of tumor necrosis factor-*α* (TNF-*α*) were measured by real-time polymerase chain reaction (PCR).

### 2.3. Diet Preparation

The high fat diet for experimental animals was a modified AIN-93 semipurified preparation [13]. The diet consisted of 33 percent energy (En%) from carbohydrates (20% sucrose and 13% starch), 16 En% from protein, and 51 En% from fats (49% lard and 2% corn oil). Normal-control diet was a 20% fat diet containing 64 percent energy (En%) from carbohydrates (10% sucrose and 54% starch), 16 En% from protein, and 20 En% from fats (49% lard and 2% corn oil). In disease-control and normal-control diets cellulose (0.6%) is included as dietary fiber. The major carbohydrate, protein, and fat sources were starch plus sugar, casein (milk protein), and lard (CJ Co., Seoul, Korea), respectively. The mixture of MB and SA was determined by our preliminary cell culture study. Mulberry water extract powder and silk amino acids were mixed to ratios of 1:3 (MS1:3) and 1:5 (MS1:5) and the mixtures were added into the high fat diet with low (0.2%) and high dosages (0.6%). The amount of mulberry extracts silk and amino acid was subtracted from casein and cellulose in the experimental diets. The nutrient compositions of the disease-control and experimental groups were equivalent.

### 2.4. Animals and Experimental Design

All experimental procedures were performed according to ARRIVE guidelines and carried out in accordance with the National Institutes of Health guide for the care and use of Laboratory animals (NIH Publications No. 8023, revised 1978). All experimental procedures were approved by the Animal Care and Use Review Committee of Hoseo University, Korea (HSIACUC-17-069). Male Sprague–Dawley rats about 11 weeks old (weighing 306 ± 20 g) were housed individually in stainless steel cages in a controlled environment (23°C and with a 12-h light/dark cycle).

The mixtures of two different ratios of MB and SA were used for animal study to verify the efficacy of improving NAFLD symptoms in rats fed a high fat diet on the basis of the HepG2 cell-based studies showing that either MB or SA alone had less efficacy to reduce fat accumulation than the two of them mixed together. We included the cell-based study in the present study. MA and SA were mixed with ratios of 1:3 (MS1:3) and 1:5 (MS1:5). The mixtures were provided in low dosage (0.2% diet; MS1:3-L and MS1:5-L) or high dosage (0.6% diet; MS1:3-H and MS1:5-H). Fifty male rats were randomly divided into five groups: 1) MS1:3-L, 2) MS1:3-H, 3) MS1:5-L, 4) MS1:5-H, and disease-control (0.6% cellulose instead of MS mixture). An additional ten rats had 20 En% fat diet with 0.6% cellulose diet as a normal-control. The low and high dosages of MS mixtures were approximately 200 and 600 mg/kg body weight/day in each rat. MB and SA were included in the assigned ratio of MB and SA. Animals were given free access to water and a high fat diet containing mulberry extracts and silk amino acids during the 12-week experimental period.

At week 11, an oral glucose tolerance test (OGTT) was conducted by oral administration of 2 g glucose/kg body weight in an overnight-fasted animal. During OGTT tail blood glucose concentrations were measured at 0, 10, 20, 30, 40, 50, 60, 70, 80, 90, and 120 mins whereas serum insulin concentrations were determined at 0, 10, 30, 60, 90, and 120 mins. Average areas under the curve (AUC) of blood glucose concentrations were calculated.

At two days after finishing the OGTT, animals had 6 h fasting and an intraperitoneal insulin tolerance test (IPITT) was performed with intraperitoneal injection of 1 IU insulin/kg bw. At two days after IPITT food was removed for 16 h and blood was collected and serum was separated by centrifugation at 4°C at 3,000 x g for 15 min. Rats were anesthetized with ketamine and xylazine mixture (100 and 10 mg/kg body weight, respectively) and human insulin (5U/kg body weight) was injected through the inferior vena cava to determine insulin signaling in the liver. Epididymal and retroperitoneal fat, uterus, and liver masses were then removed and weighed. Some liver samples were fixed with paraformaldehyde and embedded in paraffin for the histology. Serum and liver samples were then stored at -70°C for biochemical analysis.

Overnight-fasted serum glucose levels, food intakes, and body weight were measured every Tuesday at 10 am. Insulin resistance was determined using the homeostasis model assessment (HOMA) estimate of insulin resistance [HOMA-IR = fasting insulin (*μ*IU/ml) × fasting glucose (mM)/22.5] and fasting serum insulin levels [14]. The serum levels of alanine aminotransferase (ALT), aspartate aminotransferase (AST) and *γ*-glutamyl transpeptidase (*γ*-GPT), markers for liver damage, total cholesterol, HDL cholesterol, and triglyceride were measured by colorimetric methods using kits obtained from Asan Pharmaceutical company (Seoul, Korea).

### 2.5. Liver Triglyceride and Glycogen Contents

The livers were homogenized in 1.5N perchloric acid and the lysates were treated with *α*-amyloglucosidase to hydrolyze glycogen. The hydrolysates were neutralized with NaOH to pH 7.4 and centrifuged at 3000 rpm for 10 minutes and the glucose concentration measured using a glucose oxidase kit (Young Dong Pharm., Seoul, Korea). Liver glycogen was calculated from the glucose concentrations of the liver hydrolysates. Triacylglycerol was extracted with chloroform-methanol (2:1, vol/vol) from the livers and brains and resuspended in pure chloroform. After evaporating chloroform, the residues were suspended with PBS with 0.1% triton X-100 and the suspension was sonicated and boiled for 5 min. The triacylglycerol contents of the suspensions were assayed using a Trinder kit (Young Dong Pharm., Seoul, Korea).

### 2.6. Antioxidant and Inflammation Status

Lipid peroxide levels in the liver were measured using a thiobarbituric acid reactive substance (TBARS) assay kit (Cayman Chemical, Ann Arbor, Michigan, USA). The activities of antioxidant enzymes such as Cu/Zn superoxide dismutase (SOD) and glutathione (GSH)-peroxidase were measured from the lysates of the liver tissues by using colorimetry kits (Cayman Chemical, Ann Arbor, Michigan, USA and Biovision, Milpitas, CA, USA), respectively. One unit of each enzyme activity was defined as amount of SOD to inhibit the reaction by 50% and the enzyme activity was normalized by mg protein in the lysate. GSH in the liver were also determined by GSH assay kit (Sigma Aldrich, St. Louis, MO, USA).

Tumor necrosis factor-*α* (TNF-*α*) levels in serum and liver lysates were measured using ELISA kits (R & D Systems, Minneapolis, MN and Amersham Biosciences, Piscataway, NJ, USA, respectively).

### 2.7. Isolation of Liver Total RNA and Real-Time PCR

Frozen livers were powdered with a cold steel mortar and pestle and then mixed with a monophasic solution of phenol and guanidine isothiocyanate (Trizol reagent; Gibco-BRL, Rockville, MD) for total RNA extraction, according to the manufacturer's instructions. cDNA was synthesized from equal amounts of total RNA using superscript III reverse transcriptase, and polymerase chain reaction (PCR) was performed with high-fidelity Taq DNA polymerase. Equal amounts of cDNA were added to SYBR Green mix (Bio-Rad, Richmond, CA) and amplified using a real-time PCR instrument (Bio-Rad). The expression levels of the genes of interest were normalized to that of the housekeeping gene *β*-actin. To assess changes in the expression of genes related to fatty acid synthesis and oxidation in the liver, the following primers were used: sterol regulatory element-binding protein (SREBP)-1c forward 5′-GGAGCCATGG ATTGCACATT-3′, reverse 5′-AGGAAGGCTTCCAGAGAGGA-3′; acetyl CoA carboxylase (ACC) forward 5′-GGAAGATGGTGTCCCGCTCTG-3′, reverse 3′-GGGGAGATGTGCTG GGTCAT; fatty acid synthase (FAS) 5′-AGGTGCTAGAGGCCCTGCTA-3′, reverse 5′-GTGCACAGACACCTTCCCAT-3′; carnitine palmitoyl transferase (CPT)-1 5′-CTCCTGA GCAGTTACCAATGC-3′, reverse 5′-GAACCTTGGCTGCGGTAAGAC-3′; cytochrome P450 2E1 (CYP2E1) forward 5′-CTCCTCGTCATATCCATCTG-3′, reverse 5′-GCAGCC AATCAAAATGTGG-3′; TNF-*α*, forward, 5′-ACCCCCAACCTATGAAGAAA -3′ reverse, 5′-TCCACGCAAAACGGAATGAA-3′; interleukin (IL)-1*β*, forward, 5′-TTGTGGCTGT GGAGAAGCTG-3′, reverse, 5′-GCCGTCTTTCATACACAGG-3′; *β*-actin forward 5′-AAGTCCCTCACCCTCCCAAAAG-3′, reverse 5′-AAGCAATGCTGTCACCTTCCC-3′. The primers were designed to sandwich at least one intron in order to distinguish between the products derived from mRNA and genomic DNA. At least four PCR reactions per group were performed as previously described [9].

### 2.8. Immunoblot Analysis

The liver was lysed with a 20 mM Tris buffer (pH 7.4) containing 2 mM EDTA, 137 mM NaCl, 1% NP40, 10% glycerol, and 12 mM a-glycerol phosphate and protease inhibitors. After 30 min on ice, the lysates were centrifuged for 10 min at 12,000 rpm at 4°C and protein concentrations were determined using a protein assay kit (Bio-Rad). Lysate samples with equivalent protein levels (30-50 *μ*g) were directly resolved by SDS-PAGE. Immunoblotting with specific antibodies was performed against phosphorylated pAkt, Akt, pACC, ACC, and *β*-actin (Cell Signaling Technology, Beverly, MA) as previously described [15]. The intensity of protein expression was determined using Imagequant TL (Amersham Biosciences, Piscataway, NJ). These experiments were repeated four times for each group.

### 2.9. Histological Analysis

At the end of the experiments, the liver samples were fixed in 10% buffered neutral formaldehyde and embedded in paraffin wax. The 6 *μ*m thick sections from the paraffin blocks were stained with hematoxylin and eosin (H-E) and used to score the liver damage. The liver damage scores and glycogen contents were determined in two sections randomly selected from the six consecutive sections at 200X magnification. The liver damage was scored by summing each item such as the nucleus size and shape, cell size and arrangement, and the number of macrophages in histological scoring system. Each item was scored as 0 (no change), 1 (mid), 2 (moderate), and severe (3). Higher scores indicated more hepatic cell damage. In addition, glycogen contents were determined by red color intensity in periodic acid–Schiff (PAS) staining of the stomach tissues. Higher scores indicated higher glycogen contents in the liver.

### 2.10. Gut Microbiome Measured by Next Generation Sequencing (NGS)

The gut microbiome composition was measured from feces by analyzing metagenome sequencing using the NGS method. Bacterial DNA was extracted from the samples of each rat using a Power Water DNA Isolation Kit (MoBio, Carlsbad, CA) according to the manufacturer's instructions. Each library was prepared using polymerase chain reaction (PCR) products according to the GS FLX plus library prep guide. The emPCR, corresponding to clonal amplification of the purified library, was carried out using the GS-FLX plus emPCR Kit (454 Life Sciences, Branford, CT). Libraries were immobilized onto DNA capture beads. The library-beads were added to the amplification mix and oil and the mixture was vigorously shaken on a Tissue Lyser II (Qiagen, Valencia, CA) to create “micro-reactors” containing both amplification mix and a single bead. The emulsion was dispensed into a 96-well plate and the PCR amplification program was run with 16S universal primers in the Fast Start High-Fidelity PCR System (Roche, Basel, Switzerland) according to the manufacturer's recommendations. Sequencing of bacterial DNA in the feces was performed by the Macrogen Ltd. (Seoul, Korea) by a Genome Sequencer FLX plus (454 Life Sciences) as previously reported [16]. The 16S amplicon sequences were processed using Mothur v.1.36. We followed the Miseq SOP to identify the taxonomy and counts of the bacteria in each fecal sample. The picking of operational taxonomic units (OTUs) was delimited at 98% identity, which was taxonomically classified by consensus using Greengenes 13_8_99.

### 2.11. Statistical Analysis

Statistical analysis was performed using SAS, version 7.0. Results are expressed as means ± standard deviations. The disease-control, MS1:3-L, MS1:3-H, MS1:5-L, MS1:5-H, and normal-control on NAFLD were compared by one-way analysis of variance (ANOVA). Significant differences between groups were identified by Tukey's tests at P<0.05.

## 3. Results

### 3.1. Bioactive Components of Mulberry Water Extracts and Silk Amino Acids

MB contained various polyphenols such as hydroxybenzoic acid, genestic acid, rutin, luteolin, cinnamic acid, and cyanidin-3-glycosides (Table 1). SA contained 20 amino acids and it was rich in glycine, serine, alanine, valine, and aspartate (Table 1).

### 3.2. Cell Viability, TG Deposition, and Oxidative Stress of HepG2 Cell Lines Damaged by Palmitate

**Figure 1:**
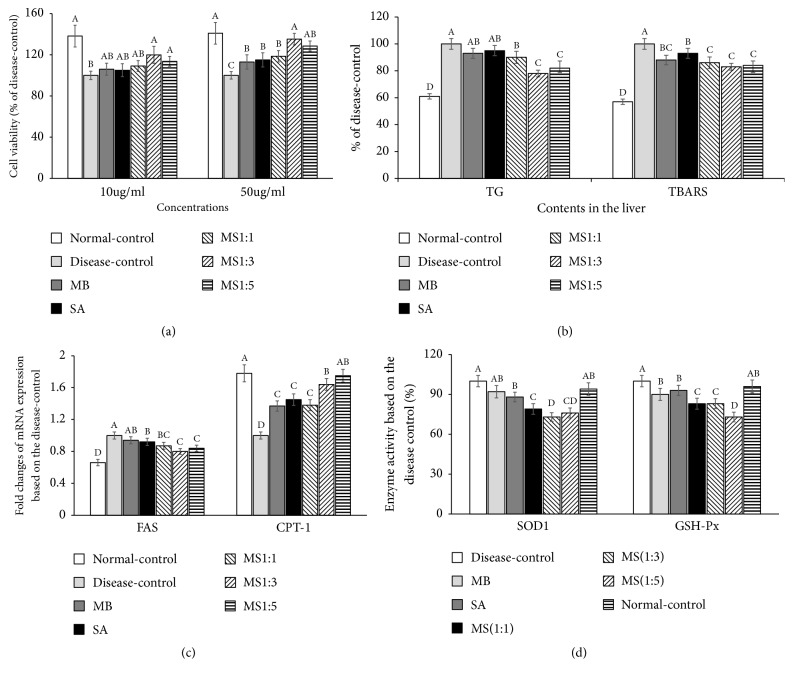
The effects of mulberry water extracts and silk amino acids on HepG2 cells administered 0.5 mM palmitate. (a) Cell viability determined by MTT assays. (b) Triglyceride and lipid peroxide contents. (c) Fold changes of fatty acid synthase (FAS) and carnitine palmitoyl transferase-1 (CPT-1) mRNA expression on the basis of disease-control. (d) Activity of superoxide dismutase 1 (SOD1) and glutathione peroxidase (GSH-Px). Each bar and error bar represents the mean ± SD (n=5). HepG2 cells were treated with 0 (vehicle), 10, or 50 *μ*g/mL mulberry extracts (MB), silk amino acids (SA) or their mixtures (MS1:2, MS1:3, and MS1:5). After 1 h of treatment, 0.5 mM emulsified palmitate with fatty acid free albumin was added to the cells and they were incubated for an additional 24 h. The assays were conducted. ^A,B,C^Bars with different letters were significantly different among the groups by Tukey test at P<0.05.

Palmitate administration (0.5 mM) suppressed cell viability in HepG2 cells and high dosage pretreatment of SA and MB and their mixture protected against the cell death by palmitate intoxication (Figure 1(a)). MS1:3 and MS1:5 improved the cell viability dose-dependently and MS1:3 protected against cell damage as much as the normal-control group in HepG2 cells.

Palmitate pretreatment increased TG accumulation in the HepG2 cells and MS1:3 and MS1:5 (50 *μ*g/mL) partially prevented the increase (Figure 1(b)). The cell damage by palmitate administration was associated with increased oxidative stress and inflammation. The MDA levels representing lipid peroxide contents were 2-fold higher in the HepG2 cells damaged with palmitate (disease-control) than in the normal-controls (Figure 1(b)). MB and SA (50 *μ*g/mL) reduced the contents of MDA induced by palmitate administration but the high dosage (50 *μ*g/mL) of MS1:3 and MS1:5 led to greater decreases than either MB or SA alone (Figure 1(b)).

The mRNA expression of FAS and CPT-1 is involved in the TG accumulation in the HepG2 cells. The fold increase of FAS was higher in the disease-control than normal-control and it was inhibited by MS1:3 and MS1:5 (50 *μ*g/mL) the most among the groups (Figure 1(c)). Opposite to FAS mRNA expression, the mRNA expression of CPT-1, a regulator of fatty acid oxidation, decreased with palmitate administration and the decrease was protected with MS1:3 and MS1:5 (50 *μ*g/mL). Decreased MDA was related to increased antioxidant enzyme activity. The SOD1 and GSH-Px activities were lower in the disease-control than the normal-control (Figure 1(d)). However, MB and SA (50 *μ*g/mL) increased SOD1 and GSH-Px activities with MB and SA alone and their mixture, MS1:3 and MS1:5, elevated their activities better than either individually (Figure 1(d)).

### 3.3. Body Weight, Food Intake, Liver Weight, and Fat Weight

Body weight was not significantly different among the groups after the 6-week treatment but it tended to be lower in the MS1:5 groups than the disease-control group regardless of dosages (Table 2). However, compared with rats in the disease-control group, the increase of body weight during 6-12 weeks of the experimental periods was suppressed at MS1:3 and MS1:5 groups in a dose dependent manner. The MS1:5-H group gained the least weight. Epididymal and retroperitoneal fat masses in the disease-control group were significantly greater compared to the normal-control (Table 2). MS1:5 treated rats showed lower epididymal and retroperitoneal mass than MS1:3 treated ones in a dose dependent manner (Table 2). Rats in the MS1:5-H group exhibited the lowest among all groups. Caloric intake was higher in the disease-control group than the low-fat group. However, caloric intake was not significantly different among the experimental groups. Thus, body weight difference was associated with different energy expenditure. Rats had about 200 and 600 mg of MS mixture per kg body weight in low and high dosages groups, respectively. MB consumption in MS1:3-H and MS1:5-H groups was 101 and 60.5 mg/ kg body weight per day, respectively, whereas SA intakes in those groups were 202 and 242 mg/ kg body weigh per day, respectively (Table 2).

### 3.4. Liver Morphometry and Histology

**Figure 2:**
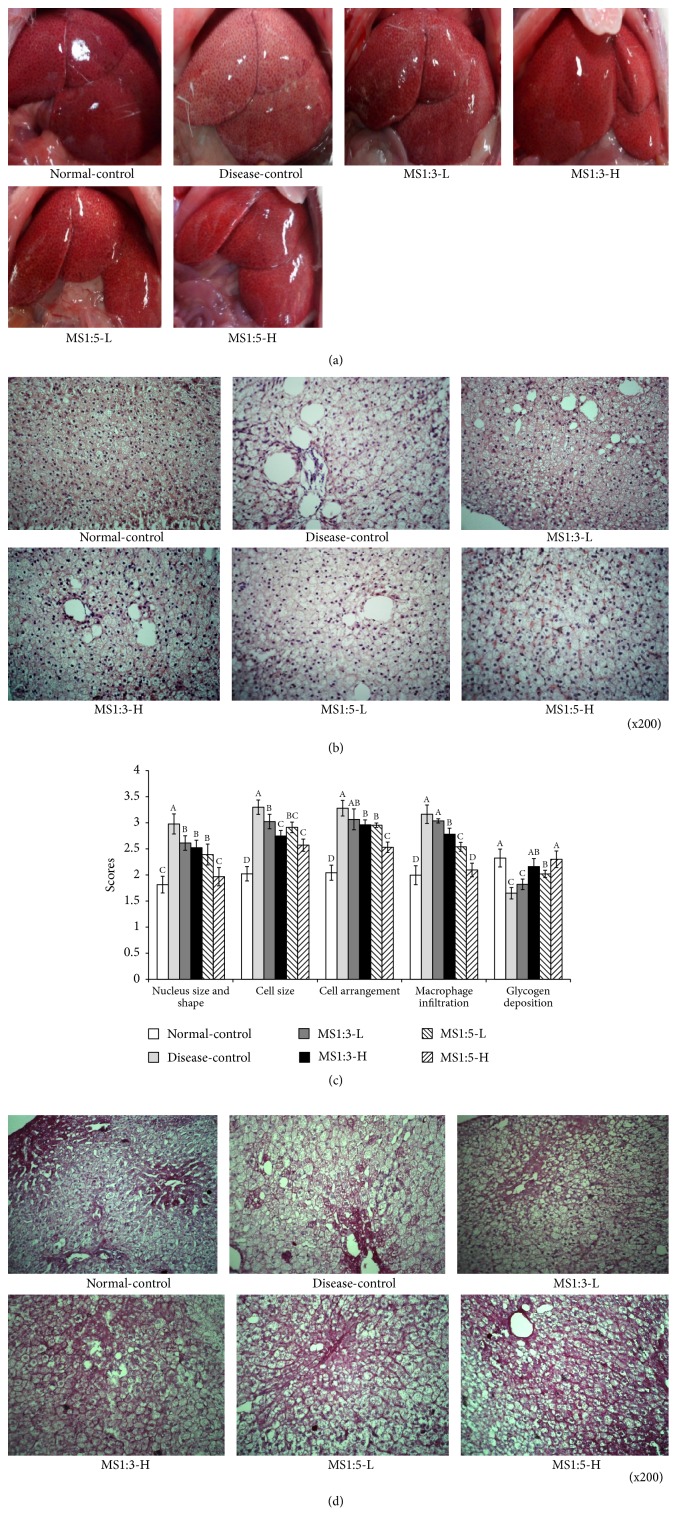
Liver morphological and histological differences. (a) The liver size and shape. (b) Staining with hematoxylin and eosin (H-E) in the liver paraffin embedded section (magnification: X200). (c) Staining with PAS in the liver paraffin embedded section (magnification: X200). (d) Scores of the nucleus and cell structure, cell arrangement, macrophage infiltration, and glycogen deposition. Each bar or dot and error bar represents the mean ± SD (n=10). Rats consumed high fat diets containing 0.2% (low dosage, -L) and 0.6% (high dosage, -H) of the mixture of mulberry extracts and silk amino acid (MS) and 0.6% cellulose (disease-control) for 12 weeks. MS1:3 and MS1:5 mixture contained 1:3 and 1:5 of mulberry extracts and silk amino acid, respectively. Sham rats were fed the same diet with the disease-control group as the normal-control. ^A,B,C^Bars with different letters were significantly different among the groups by Tukey test at P<0.05.

After 12 weeks of treatment, the liver was enlarged and fat accumulation increased in the disease-control compared to the normal-control, and MS1:3-H and MS1:5-H prevented the increase in liver size and fat accumulation as shown in Figure 2(a). Liver weight was higher in the disease-control than the low-fat diet group. MS1:3 and MS1:5 treated rats had dose-dependently lower liver weights and MS1:5 treatment lowered the liver weight more than MS1:3 (Table 2).

H&E histology section showed that the morphology of the liver tissue was markedly changed in rats of the disease-control group compared to those of the normal-control group (Figures 2(b) and 2(d)). Cell arrangement was irregular and cells were enlarged in rats fed a high fat diet compared to the normal-control (Figures 2(b) and 2(d)). Ballooning degeneration, a process of liver parenchymal cell death caused by enlarged cell size, developed in the disease-control group. MS1:3 and MS1:5 suppressed the ballooning degeneration and MS1:5 was more effective for preventing liver damage. In addition, macrophage infiltration was higher in the disease-control than normal-control and MS1:3 and MS1:5 inhibited macrophage infiltration (Figures 2(b) and 2(d)). Thus, MS1:3 and MS1:5 suppressed liver damage induced by high fat diets and MS1:5-H is most effective in preventing the liver tissue damage. MS1:3 and MS1:5 alleviated the hepatic histological alteration and MS1:5-H displayed the most effective alleviation of histological changes by a high fat diet.

PAS staining shows glycogen deposition and cell connective tissues in the liver (Figures 2(c) and 2(d)). Rats fed a high fat diet reduced the red staining indicating glycogen and cellular connective tissues compared to those in the normal-control group. MS1:3-H and MS1:5-H showed the suppression of red staining compared to the disease-control, indicating that they prevented the cellular damage caused by a high fat diet (Figures 2(c) and 2(d)).

### 3.5. Liver Damage and Oxidative Stress

Rats in the disease-control group showed significantly high levels of AST, ALT, and *γ*-GPT compared to those in the low-fat diet group. Rats administered MS1:3 and MS1:5 had significantly lower serum ALT, AST, and *γ*-GPT, in a dose-dependent manner, in comparison to the disease-control (Table 3). Serum TNF-*α* levels, an index of inflammation, were lower in the rats administered MS1:3-H and MS1:5-H than the disease-control rats. MS1:5-H lowered serum TNF-*α* than MS1:3-H.

Decreased serum levels of AST, ALT, and *γ*-GPT by MS1:3 and MS1:5 administration indicate the alleviation of liver damage by a high fat diet (Table 3). The contents of MDA, an index of lipid peroxide level, in the liver were significantly higher in the disease-control group than the low-fat diet group. Hepatic MDA levels were significantly lower in the MS1:3 and MS1:5 than the disease-control and were lowest in the MS1:3-H among all groups (Table 3). Lipid peroxide levels with a high fat diet are mediated by antioxidant enzymes such as SOD and GSH-Px, and GSH-Px uses GSH as a supplier to remove free electron that induce oxidative stress [17]. Rats in the disease-control group exhibited higher hepatic SOD and GSH-Px activities but lower GSH levels than the normal-control (a low-fat diet). This indicated that MS1:5 inhibited the decrease of hepatic SOD and GSH-Px activities and GSH levels as much as the normal-control (Table 3). Rats having MS1:3-H diet suppressed the decrease of hepatic SOD and GSH-Px activities and the increase of hepatic GSH the most by a high fat diet (Table 3). MS1:5 administration improved antioxidant enzyme activities better than the normal-control. In addition to the antioxidant enzyme activities, CYP2E1, an index of oxidant and toxic substance levels, CYP2E1 mRNA expression was lower in the normal-control than the disease-control, indicating a high fat diet increased oxidative substances (Table 3). CY2E1 mRNA expression was suppressed by MS1:3 and MS1:5 dose-dependent manner, and MS1:3-H inhibited the mRNA levels of CYP2E1 the most among the treatments (Table 3).

**Figure 3:**
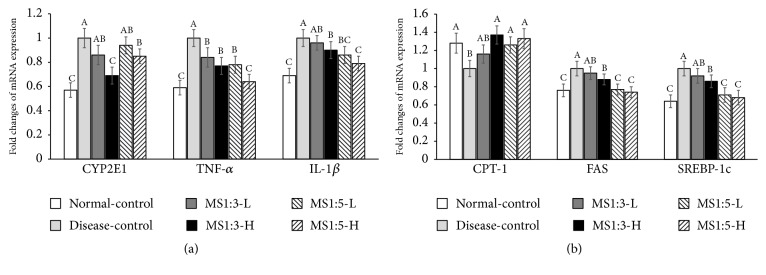
Relative gene expression related to fatty acid utilization and synthesis, oxidative stress, and inflammation, and hepatic insulin signaling. (a) Fold changes of mRNA expression of genes related to fatty acid synthesis. (b) Fold changes of mRNA expression of genes related to oxidative stress and inflammation. Each bar or dot and error bar represents the mean ± SD (n=10). Rats consumed high fat diets containing 0.2% (low dosage, -L) and 0.6% (high dosage, -H) of the mixture of mulberry extracts and silk amino acid (MS) and 0.6% cellulose (disease-control) for 12 weeks. MS1:3 and MS1:5 mixture contained 1:3 and 1:5 of mulberry extracts and silk amino acid, respectively. ^A,B,C^Bars with different letters were significantly different among the groups by Tukey test at P<0.05.

Hepatic inflammation states in the liver were shown with fold changes of TNF-*α* and IL-1*β* mRNA expression. TNF-*α* and IL-1*β* RNA expression exhibited the normal-control compared to the disease-control. MS1:3 and MS1:5 dose-dependently showed the inhibition of TNF-*α* mRNA expression in comparison to the disease-control and MS1:5-H decreased the expression as much as the normal-control (Figure 3(a)). As similar to TNF-*α* expression, IL-1*β* mRNA expression was much lower in the normal-control than in the disease-control. MS1:5 lowered the increase of IL-1*β* mRNA expression to less than the disease-control but its mRNA expression was higher than the normal-control (Figure 3(a)).

### 3.6. Lipid Profile and Hepatic Triglyceride Deposition

Serum total cholesterol levels were not significantly different among the group. However, high fat fed rats exhibited significantly higher levels of serum LDL cholesterol than low-fat fed rats (Table 4). MS1:3 and MS1:5 inhibited the increase of serum LDL levels in rats fed a high fat diet in a dose-dependent manner (Table 4). Furthermore, serum HDL levels were significantly lower in the disease-control than the normal-control and MS1:3-H and MS1:5-H increased the levels. Serum triglyceride levels were not significantly different between the disease-control and normal-control groups. However, MS1:5 administration lowered serum TG levels to less than the disease-control group (Table 4). Thus, high dosage administration of MS1:3 and MS1:5 improved lipid profiles in rats fed a high fat diet. The main problem with NAFLD is the deposition of triglyceride in the liver. Triglyceride deposition in the liver was about 2-fold higher in the disease-control group than the normal-control group (Table 4). MS1:3 and MS1:5 suppressed the triglyceride deposition in the liver and MS1:5-H inhibited the deposition the most although the inhibition did not suppress the accumulation as much as the normal-control group (Table 4). By contrast, glycogen accumulation was lower in the rats fed a high fat diet (disease-control group) than those fed a low-fat diet (normal-control group). MS1:5 promoted glycogen accumulation better than MS1:3 and normal-control groups (Table 4).

TG deposition is the net of TG oxidation and TG synthesis. The rate-limiting regulatory step of fatty acid oxidation is the CPT-1 facilitated transport of fatty acids across the mitochondria inner membrane. The mRNA CPT-1 expression is a primary regulatory step of fatty acid oxidation. The CPT-1 mRNA expression of the disease-control was decreased compared to the normal-control (Figure 3(b)). MS1:3-H, MS1:5-L, and MA1:5-H increased the CTP-1 mRNA expression compared to the disease-control and the increase of CPT-1 mRNA was similar to the normal-control (Figure 3(b)). FAS and SREBP-1c also regulate fatty acid synthesis. The mRNA expression of FAS was significantly higher in the disease-control group than the normal-control group. MS1:5-L and MS1:5-H caused a decrease in FAS mRNA expression in comparison to the disease-control (Figure 3(b)). SREBP-1c expression was higher in the disease-control group than the normal-control group. MS1:3-H, MS1:5-L, and MS1:5-H lowered the mRNA expression of SREBP-1c to less than the disease-control and MS1:5 decreased it the most among the treatments, but it was higher than the disease-control (Figure 3(b)).

### 3.7. Glucose Metabolism

**Figure 4:**
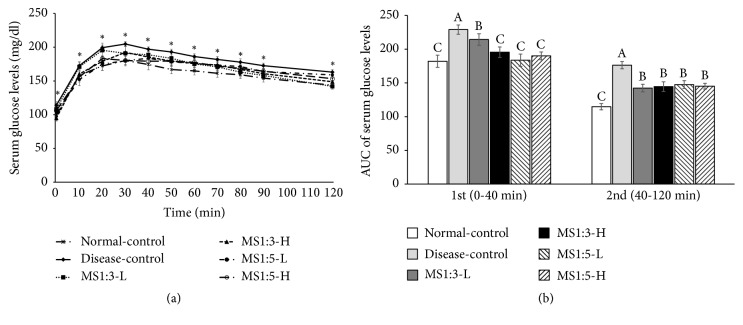
Serum glucose levels during oral glucose tolerance test. (a) Changes of serum glucose concentrations in 16-h fasted rats after oral challenge of 2 g glucose/kg body weight. (b) Area under the curve (AUC) of serum glucose calculated in the first (0-40 min) and second phases (40-120 min). Each bar or dot and error bar represents the mean ± SD (n=10). Rats consumed high fat diets containing 0.2% (low dosage, -L) and 0.6% (high dosage, -H) of the mixture of mulberry extracts and silk amino acid (MS) and 0.6% cellulose (disease-control) for 12 weeks. MS1:3 and MS1:5 mixture contained 1:3 and 1:5 of mulberry extracts and silk amino acid, respectively. ^*∗*^Significantly different among the groups at P<0.05. ^A,B,C^Bars with different letters were significantly different among the groups by Tukey test at P<0.05.

As shown in Figure 4(a), serum glucose levels were consistently higher in the disease-control group than the normal-control group during OGTT, indicating that the rats fed a high fat had glucose intolerance. MS1:3-L and MS1:3-H dose-dependently decreased serum glucose levels during the first part of OGTT but the decrease of MS1:3-L was less than MS1:5-L. MS1:5-L and MS1:5-H had a similar decrease of serum glucose levels during the 1^st^ phase of OGTT and the decrease was similar to the normal-control (Figure 4(a)). Serum glucose levels were much higher in the disease-control group than normal-control group during the 2nd phase of OGTT and MS1:3 and MS1:5 inhibited the increase in rats fed a high fat diet to maintain serum glucose levels to as much as the normal-control group (Figure 4(a)). Area under the curve (AUC) of 1^st^ and 2^nd^ phase during OGTT reflected serum glucose levels (Figure 4(b)). MS1:5 exhibited similar ACU of 1^st^ and 2^nd^ phases as the normal-control group (Figure 4(b)).

**Figure 5:**
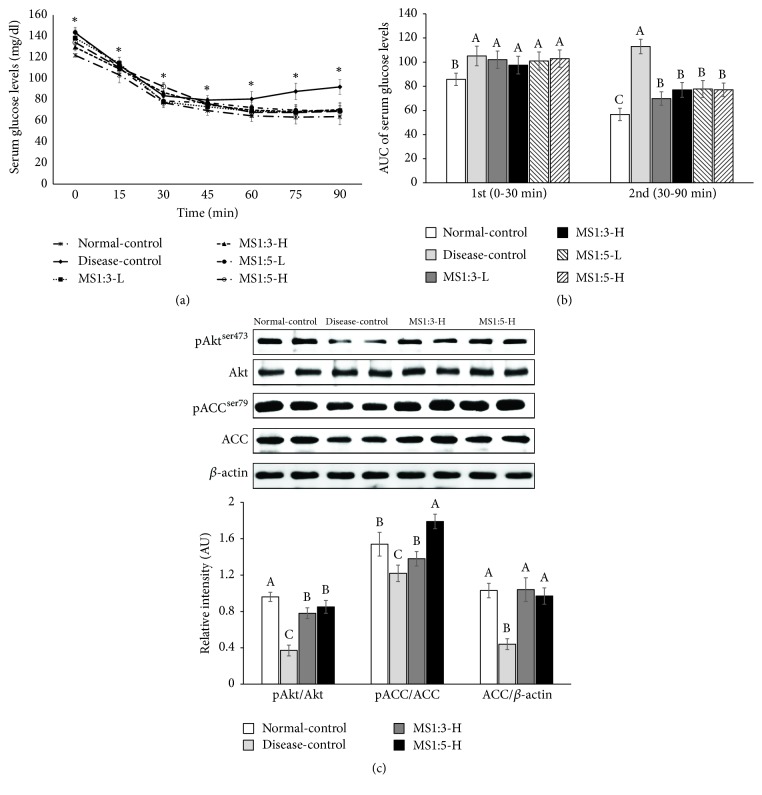
Changes of serum glucose levels during insulin tolerance test and hepatic insulin signaling. (a) Changes of serum glucose concentrations after 1 U insulin/kg body weight (BW) into subcutaneous injection after 6h food deprivation. (b) Area under the curve (AUC) of serum glucose levels calculated in the first (0-30 min) and second phases (30-90 min). (c) Hepatic insulin signaling pathway injecting 0.75 U insulin /kg bw in 16h fasting animal at the end of experiment. Each bar or dot and error bar represents the mean ± SD (n=10). Rats consumed high fat diets containing 0.2% (low dosage, -L) and 0.6% (high dosage, -H) of the mixture of mulberry extracts and silk amino acid (MS) and 0.6% cellulose (disease-control) for 12 weeks. MS1:3 and MS1:5 mixture contained 1:3 and 1:5 of mulberry extracts and silk amino acid, respectively. ^*∗*^Significantly different among the groups at P<0.05. ^A,B,C^Bars with different letters were significantly different among the groups by Tukey test at P<0.05.

After 6 h of food deprivation, the changes in serum glucose levels after intraperitoneal insulin tolerance test (IPITT) are shown in Figure 5(a). Serum glucose levels were higher in the disease-control group than the normal-control group. All rats markedly decreased serum glucose levels until 30-45 min after insulin injection and after 30-45 min serum glucose levels gradually decreased or were maintained in most groups except the disease-control group (Figure 5(a)). However, rats in the disease-control group had a rebounce of serum glucose levels after 45 min (Figure 5(a)). AUC of the first part (0-30 min) was higher in the disease-control group than the normal-control group during IPITT but it was not significantly different among the treatments (Figure 5(b)). AUC of the 2^nd^ part was much higher in the disease-control than the normal-control and MS1:3 and MS1:5 reduced the AUC but it was higher than the normal-control (Figure 5(b)).

### 3.8. Insulin Signaling in the Liver

Akt was less phosphorylated in the disease-control than the normal-control, indicating that insulin signaling was attenuated in the rats fed a high fat diet. MS1:3-H and MS1:5-H suppressed the decrease of its phosphorylation (Figure 5(c)). ACC protein levels were lower in the disease-control group than the normal-control group whereas MS1:3-H and MS-1:5-H increased ACC protein (Figure 5(c)). The phosphorylation of ACC was lower in the normal-control than the disease-control and MS1:3 and MS1:5 elevated the phosphorylation of ACC. This indicated that increased phosphorylation of ACC decreased ACC activity to make malonyl CoA from acetyl CoA and reduced the substrates for fatty acid synthesis. MS1:3-H potentiated its phosphorylation the most among the treatments (Figure 5(c)). Thus, MS1:3-H and MA1:5-H potentiated insulin signaling in the liver.

### 3.9. Gut Microbiome

**Figure 6:**
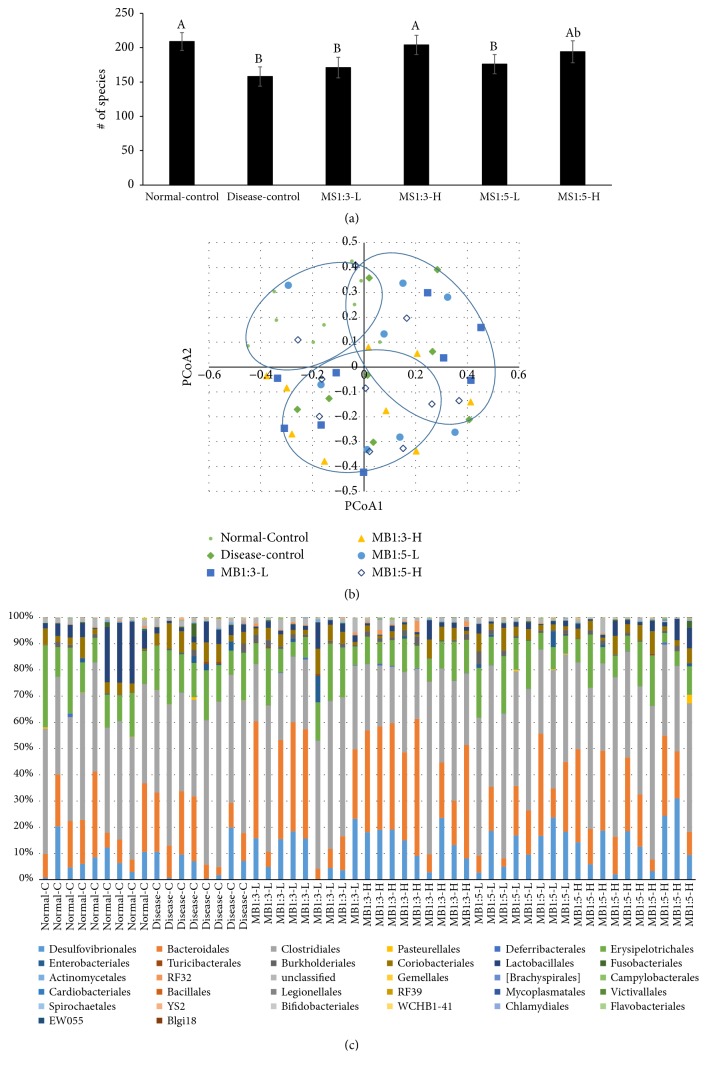
The profiles of gut microbiome by NGS analysis of gut microbial DNA. (a) The richness of gut microbiomes. (b) The fecal bacterial community by principal coordinate analysis (PCoA). (c) Proportion of taxonomic assignments [family] for gut microbiomes. Rats consumed high fat diets containing 0.2% (low dosage, -L) and 0.6% (high dosage, -H) of the mixture of mulberry extracts and silk amino acid (MS) and 0.6% cellulose (disease-control) for 12 weeks. MS1:3 and MS1:5 mixture contained 1:3 and 1:5 of mulberry extracts and silk amino acid, respectively. ^A,B,C^Bars with different letters were significantly different among the groups by Tukey test at P<0.05.

Fatty liver is associated with changes in the gut microbiome. Bacterial richness, determined by the total number of operational taxonomic units (OUTs), was lower in the disease-control group than the normal-control group (Figure 6(a)). The decrease in bacterial richness was prevented by MS1:3-H similar to the normal-control (Figure 6(a)).

Differences in the microbiota community were analyzed by the principal coordinate analysis (PCoA) plots to show the clustering of gut bacterial communities among the groups (Figure 6(b)). An unweighted distance-based analysis of molecular variance (AMOVA) was used to assess the statistical significance of the spatial separation among the groups in the PCoA plots (Figure 6(b)). The AMOVA test exhibited significant differences in the fecal bacterial communities among the groups (P=0.01). Fecal bacterial communities of the disease-control and normal-control groups were significantly separated by the disease-control and normal-control groups but only MA1:3-H showed a significant separation from not only the disease-control group (P=0.02; Figure 6(b)) but also the normal-control group (P=0.04). Other treatments were not separated from the disease-control and normal-control group (Figure 6(b)). This indicated that the gut microbiome might be altered by the nonalcoholic hepatic steatosis and treatments. Among the components of MS, silk amino acid effects might be minimal.

The bacterial distribution was different among the groups at the order level (Figure 6(c)). The major bacteria were* Bacteroidales*,* Clostridiales*,* Erysipelotrichales*,* Desulfovibrionales*,* Lactobacillales*, and* Deferribacterales *at the order level and some others were also present (Figure 6(c)). Normal-control group (0.39 ± 0.07 %) exhibited higher levels of the ratio of* Bacteroidales* and* Clostridiales* than the disease-control group (0.24 ± 0.06 %). The MS1:3-L (0.65 ± 0.11 %) and MS1:3-H (0.92 ± 0.16 %) treatment increased the ratio in a dose-dependent manner in comparison to the disease-control. MS1:5 increased the ratio but not as much as MS1:3 (Figure 6(c)). The normal-control (10.2 ± 2.4 %) had* Lactobacillales* much higher than the disease-control (3.6 ± 0.9 %) and the treatments did not change the levels of* Lactobacillales*. MS1:5-H also reduced* Turicibacterales*, which are known to induce inflammation by reducing toll-like receptor 4/TNF-*α* signaling [18]. Thus, MS1:3-H improved the gut microbiota to alleviate nonalcoholic hepatic steatosis and MS1:5 partly alleviated gut dysbiosis. Silk amino acids did not influence gut microbiome as much as mulberry extracts in rats fed a high fat diet.

## 4. Discussion

NAFLD is a pathological accumulation of TG in the inside of liver cells [19]. NAFLD risk factors include various metabolic diseases such as obesity, type 2 diabetes, and dyslipidemia [19]. The reduction of TG deposition is a potential target for treating NAFLD. We investigated the efficacy of the mixture of mulberry extracts and silk amino acids for decreasing hepatic TG and the possible mechanism involved in alleviating fatty liver by high fat diets. We found that the rats fed a high fat diet (disease-control group) developed insulin resistance in concert with increased body weight gain, visceral fat mass, and liver triglyceride content. MS1:3-H and MS1:5-H were found to reduce TG accumulation in the liver, which was associated with decreased oxidative stress and inflammation. MS1:3-H alleviated oxidative stress while elevating the ratio of* Bacteroidales* and* Clostridiales* in the cecum better than MS1:5-H, but MS1:5-H suppressed inflammation and lowered hepatic triglycerides better, which prevented the liver tissue damage. Thus, MS1:3-H and MS1:5-H prevented nonalcoholic steatosis by partly different pathways.

NAFLD animal models are made with (1) genetic modification and (2) dietary (high fructose or high fat diet) or (3) pharmacological treatments [20]. NAFLD is accompanied with hepatic cell damage induced by increased oxidative stress and inflammation leading to increased fat deposition in the liver [20]. In the present study a high fat diet (50% lard and 2.5% cholesterol) as a dietary modification was used. The characteristics of NAFLD (including hepatomegaly, hepatocyte ballooning, steatosis, and steatohepatitis) were observed in the rats in the disease-control group. They were associated with increased oxidative stress and inflammation. MS1:3 and MS1:5 partly prevented the symptoms of NAFLD. MS1:3 and MS1:5 suppressed oxidative stress and inflammation compared to the disease-control, and MS1:5 retained the normal characteristics of hepatic tissues. MS1:5-H also suppressed the TG deposition in the liver and potentiated glycogen accumulation the most in the present study. MB has been reported to alleviate liver damage induced by different triggers such as acrylamide and carbamate while reducing oxidative stress [13, 14]. In previous studies silk amino acids have not been studied for preventing oxidative stress, but they have been reported to ameliorate inflammation [21]. Thus, the mixtures of MB and SA had much better activity to protect against NAFLD.

TG deposition in the liver is initially associated with increased hepatic insulin resistance [22, 23]. Insulin resistance is also involved in the severity of NAFLD [23]. Insulin resistance can be estimated by several methods, and in the present study it was measured by HOMA-IR, IPITT, and hepatic insulin signaling. The animal model showed much higher insulin resistance measured by HOMA-IR and IPITT than the normal-control, and both MS1:3-H and MS1:5-H inhibited the progression of insulin resistance. The lower HOMA-IR was not significantly different between MS1:3 and MS1:5 and the decrease was as low as the normal-control. Consistent with the present study, mulberry anthocyanins have been demonstrated to suppress body fat accumulation and improve insulin sensitivity in high fat diet fed mice [24]. It has also been shown that hepatic TG deposition was much higher in the disease-control and MB reduced the TG accumulation as much as the normal-control [24].

In the present study, MS1:3-H and MS1:5-H potentiated phosphorylation of Akt and ACC in the liver and MS1:5-H stimulated the phosphorylation more than MS1:3-H. Thus, MS1:3 and MS1:5 both alleviated insulin resistance. Mulberry ethanol extracts (rich in anthocyanins) have been reported to ameliorate insulin resistance by potentiating the phosphoinositide 3-kinase *⟶*Akt pathway in the liver [15, 25]. However, anthocyanins are heat sensitive and prone to oxidation [26], so in the present study, mulberry was extracted in 60°C water to prevent the degradation of anthocyanins and it included low concentrations of anthocyanins. As previously reported [27, 28], the major bioactive compounds in the mulberry extracts were listed as quercetin, hydroxybenzoic acid (3.66 ± 0.18 mg/g extract), rutin (8.6 ± 0.00 mg/g extract), cyanidin-3-glucoside (6.45 ± 0.03 mg/g extract), and cinnamic acid (0.41 ± 0.06 mg/g extract). Silk amino acids may have the potential to reduce insulin resistance since it has been reported to have antidiabetic activity by increasing *β*-cell mass [29].

The molecular mechanisms involved in the progression of NAFLD to inflammation, hepatocellular injury, and fibrosis are not completely understood [30]. The primary insult is lipid accumulation in the hepatocytes and it is accompanied by inflammation due to increasing proinflammatory mediators. An imbalance between lipid deposition and removal results in the accumulation of TG and NEFA. Increased NEFA suppresses *β*-oxidation of fatty acid to make energy and promotes TG synthesis in the liver [31]. Chronic elevation of NEFA induces insulin resistance and this process makes a vicious cycle to induce NAFLD. Furthermore, increased insulin resistance stimulates lipolysis and increases TNF-*α* production in the adipocytes [32]. Wu et al. [28] also showed that mulberry juice decreased TG deposition in the liver. Silk amino acid is reported to have anti-inflammatory activity [21]. The MB and SA mixture had a synergistic activity to improve the inflammation and reduce insulin resistance. Thus, MS1:5-H might inhibit the progression of NAFLD to NASH.

In addition to the inflammation, abundant free fatty acids cause lipotoxicity via the induction of ROS release [33]. CYP2E1, a member of the oxidoreductase cytochrome family, oxidizes various small toxic substrates including xenobiotics, ethanol, and fatty acids [34]. Superoxide anions, very potent ROS, are produced as a byproduct of the CYP2E1-mediated metabolism. CYP2E1 knockout mice exhibit a marked protection against high fat diet-induced insulin resistance with enhanced adipose tissue glucose uptake, insulin suppression of hepatic glucose output, and decreased proinflammatory cytokines from adipose tissues [35]. CYP2E1 expression and activity in the liver is increased in humans and in animal models of NAFLD [34], as shown in the present study. MS1:3-H resulted in CYP2E1 expression in the liver as low as the normal-control, and MS1:5-H decreased its expression but it was higher than MS1:3-H. Liver damage decreases the oxidation of fatty acids and packaging and exporting of VLDL from the hepatocytes [31]. In the present study the levels of lipid peroxides in the liver were higher in the disease-control group than the normal-control group although hepatic SOD and GSH-peroxide activity was higher in the disease-control group than the normal-control group, as shown in other reports [36]. MS1:3 and MS1:5 decreased the lipid peroxide content and decreased hepatic GSH levels in comparison to the disease-control group and they also reduced the activities. MS1:5-H reduced their activities similar to the normal-control group. Contrary to antioxidant enzyme activities, hepatic GSH content, an index of antioxidant state, was lower in the disease-control group than the normal-control group. MS1:3-H exhibited a higher hepatic GSH content. Antioxidants can suppress the depletion of GSH [37] and MS1:3-H was shown to reduce hepatic oxidative stress by TG accumulation the most among the treatments.

Previous studies have demonstrated that mulberry extracts contain phenolic compounds, ascorbic acid, and multiple minerals with various biological activities including antioxidant, antidiabetic, and hypolipidemic activities [38, 39]. However, anthocyanins, one category of bioactive components in mulberries, are known to be partly degraded by heating during its water extraction and they are easily converted into other phenolic compounds by bacteria during digestion [38]. In the present study the water extraction of mulberry was processed at 60°C to reduce the loss of bioactive compounds [26]. During digestion, phenolic compounds including anthocyanins are converted into 4 flavonoids such as quercetin rhamnosyl hexoside hexoside, quercetin rhamnosyl hexoside, quercetin hexoside, and kaempferol rhamnosyl hexoside, which may be why digested mulberry reduces palmitate induced lipotoxicity by decreasing oxidative stress [38]. Those results suggested that mulberry extracts may ameliorate nonalcoholic fatty liver, which is consistent with the present study. The silk protein, sericin, is reported to exert antidiabetic activity by promoting pancreatic *β*-cell mass in db/db mice [29]. In addition, silk amino acid administration protects against tissue damage from physical exercise [40]. Thus, silk amino acids may improve tissue regeneration and it may promote healing of hepatic tissue damaged by inflammation caused by a high fat diet. The present study demonstrated that both MS1:3-H and MS1:5-H improved NAFLD, but their main mechanisms were somewhat different. MS1:3-H reduced oxidative stress better than MS1:5-H whereas MS1:5-H promoted anti-inflammation and protected against the damage of hepatic tissues than MS1:3-H.

In addition to metabolic disorders, inflammation is also influenced by gut microbiota; and the present study showed that gut microbiota was altered in rats with NAFLD and that diet also changed the gut microbiome. Previous research found that Mulberry extract (0.3g/kg bw) did not increase* Bacteroidales* and decrease* Clostridiales,* although dandelion extracts increased* Bacteroidales *and decreased* Clostridiales* in rats with alcoholic hepatic steatosis [9]. The ratio of* Bacteroidales* and* Clostridiales* was lower in the normal-control group than the disease-control group. The MS1:3-L and MS1:3-H treatments increased the ratio in a dose-dependent manner.* Lactobacillales* was much higher in the normal-control than the disease-control and the treatments did not change the levels of* Lactobacillales*. Thus, MS1:3-H improved the gut microbiota and the alteration might be associated with alleviating nonalcoholic hepatic steatosis.

## 5. Conclusions

A high fat diet (51 energy % fat, 2.5% cholesterol, and 22.7% sucrose) caused NAFLD which was associated with oxidative stress, inflammation, and insulin resistance in male rats. MS1:3-H and MS1:5 improved the symptoms of NAFLD by reducing hepatic TG deposition and hepatic cell damage. MS1:3-H reduced oxidative stress better than MS1:5-H but MS1:5-H decreased inflammation and prevented the hepatic cell damage better than MS1:3-H. MS1:3-H improved gut microbiome richness and was separated from the disease-control group. Thus, a combination of MS1:3-H and MS1:5-H might be a safe and effective therapeutic agent for preventing or alleviating NAFLD.

## Figures and Tables

**Table 1:** Contents of total phenols and flavonoids and bioactive components in water extracts of mulberry.

Mulberry	Total polyphenols	Total flavonoids	Rutin	Hydroxybenzoic acid	Cyanidin-3-glucoside
	35.1 ± 0.7	24.8 ± 0.6	8.82 ± 0.06	3.71 ± 0.14	6.51 ± 0.04

Silk amino acid	Glycine	alanine	Serine	Valine	Aspartate
	8.40 ± 0.43	7.09 ± 0.51	2.75 ± 0.12	0.71 ± 0.03	0.48 ± 0.02

Values represent means ± standard deviation (n=3).

**Table 2:** Energy metabolism and mulberry and silk amino acid intake.

	Normal-control (n=10)	Disease-control (n=10)	MS1:3-L (n=10)	MS1:3-H (n=10)	MS1:5-L (n=10)	MS1:5-H (n=10)
Body weight at 6 weeks (g)	451 ± 26	453 ± 31	458 ± 16	454 ± 25	451 ± 26	455 ± 17
Body weight at 12 weeks (g)	474 ± 35^C^	553 ± 33^A^	530 ± 33^AB^	523 ± 36^AB^	515 ± 29^B^	509 ± 24^B^
Weight gain during 6-12 weeks (g)	23.3 ± 1.8^E^	100.5 ± 5.6^A^	72.4 ± 4.2^B^	69.2 ± 4.5^B^	64.2 ± 4.3^C^	56.6 ± 4.5^D^
Epididymal fat pads (g)	6.7 ± 1.2^C^	9.9 ± 0.9^A^	10.1 ± 0.9^A^	9.2 ± 1.4^AB^	8.5 ± 0.9^B^	7.3 ± 1.1^C^
Retroperitoneal fat (g)	9.0 ± 1.3^C^	13.5 ± 1.8^A^	12.6 ± 0.8^AB^	12.2 ± 1.3^AB^	11.9 ± 1.4^B^	9.8 ± 1.2^C^
Caloric intake (kcal/day)	86.8 ± 6.9^B^	108 ± 6.9^A^	106 ± 8.6^A^	105 ± 8.0^A^	103 ± 7.7^A^	106 ± 8.4^A^
MB intake (mg/kg bw/ day)	0 ± 0^C^	0 ± 0^C^	63.4 ± 4.7^C^	193 ± 15^A^	39.6 ± 3.3^D^	119 ± 9.0^B^
SA intake (mg/kg/ bw/day)	0 ± 0	0 ± 0	127 ± 9.1^D^	386 ± 27^B^	160 ± 13^C^	475 ± 35^A^
Liver weight (weight %)	4.4 ± 0.3^D^	5.8 ± 0.4^A^	5.5 ± 0.3^AB^	5.4 ± 0.2^B^	5.2 ± 0.2^BC^	5.1 ± 0.2^C^

Values are means ± standard deviation. Rats consumed high fat diets containing 0.2% (low dosage, -L) and 0.6% (high dosage, -H) of the mixture of mulberry extracts and silk amino acid (MS) and 0.6% cellulose (disease-control) for 12 weeks. MS1:3 and MS1:5 mixture contained 1:3 and 1:5 of mulberry extracts and silk amino acid, respectively. Sham rats were fed the same diet with the disease-control group as the normal-control.

^A,B,C,D,E^Means on the same row with different letters were significantly different by Tukey test at p < 0.05.

**Table 3:** Liver damage index and antioxidant enzyme activity in the liver.

	Normal-control (n=10)	Disease-control (n=10)	MS1:3-L (n=10)	MS1:3-H (n=10)	MS1:5-L (n=10)	MS1:5-H (n=10)
Serum AST (U/L)	80.4 ± 5.7^BC^	94.1 ± 6.8^A^	83.5 ± 6.2^B^	77.0 ± 5.6^C^	84.8 ± 5.9^B^	75.0 ± 5.1^C^
Serum ALT (U/L)	26.1 ± 3.5^C^	41.7 ± 6.6^AB^	36.6 ± 4.3^B^	28.8 ± 5.3^C^	42.7 ± 58^A^	33.2 ± 4.8^BC^
Serum *γ*-GPT (U/L)	48.4 ± 3.7^D^	71.3 ± 6.2^A^	64.7 ± 5.9^B^	55.1 ± 5.3^C^	62.7 ± 5.6^B^	51.6 ± 5.8^CD^
Serum TNF-*α* (pg/mL)	23.4 ± 3.0^E^	59.8 ± 5.1^A^	45.3 ± 5.3^B^	36.4 ± 4.2^C^	34.5 ± 3.9^C^	28.6 ± 3.1^D^
Hepatic MDA (nmol/mg protein)	0.64 ± 0.05^D^	0.93 ± 0.08^A^	0.79 ± 0.07^B^	0.71 ± 0.07^C^	0.84 ± 0.07^B^	0.78 ± 0.07^B^
Hepatic SOD (U/mg protein)	39.8 ± 3.5^A^	27.3 ± 2.8^C^	31.1 ± 3.6^BC^	36.4 ± 3.7^AB^	26.7 ± 3.1^C^	30.5 ± 3.2^BC^
Hepatic GSH peroxide (U/mg protein)	85.1 ± 6.3^A^	65.4 ± 4.7^C^	72.6 ± 4.9^B^	78.3 ± 5.5^AB^	69.7 ± 5.3^BC^	73.4 ± 5.1^B^
Hepatic GSH (umol/g protein)	24.9 ± 2.3^B^	22.8 ± 2.1^C^	25.1 ± 2.2^B^	27.7 ± 2.1^A^	23.5 ± 2.5^BC^	24.8 ± 2.2^B^
Hepatic TNF-*α* (pg/g tissue)	5.7 ± 0.6^D^	9.2 ± 0.9^A^	8.6 ± 0.9^AB^	7.9 ± 0.8^B^	8.2 ± 0.8^B^	7.1 ± 0.8^C^

Values are means ± standard deviation. AST, aspartate aminotransferase; ALT, alanine aminotransferase; *γ*-GPT, *γ*-glutamyl transferase; TNF-*α*, tumor necrosis factor-*α*; MDA, malondialdehyde; SOD, superoxide dismutase; GSH, glutathione. Values are means ± standard deviation. Rats consumed high fat diets containing 0.2% (low dosage, -L) and 0.6% (high dosage, -H) of the mixture of mulberry extracts and silk amino acid (MS) and 0.6% cellulose (disease-control) for 12 weeks. MS1:3 and MS1:5 mixture contained 1:3 and 1:5 of mulberry extracts and silk amino acid, respectively. Sham rats were fed the same diet with the disease-control group as the normal-control.

Sham rats were fed the same diet with the disease-control group as the normal-control.

^A,B,C,D,E^Means on the same row with different letters were significantly different by Tukey test at p < 0.05.

**Table 4:** Lipid profile and glucose and insulin in the circulation, HOMA-IR and hepatic triglyceride, and glycogen deposition.

	Normal-control (n=10)	Disease-control (n=10)	MS1:3-L (n=10)	MS1:3-H (n=10)	MS1:5-L (n=10)	MS1:5-H (n=10)
Serum total cholesterol	94.8 ± 5.7	96.9 ± 7.9	95.4 ± 6.5	92.5 ± 6.4	97.4 ± 7.9	92.0 ± 7.2
Serum HDL-C	23.1 ± 1.6^B^	18.6 ± 1.2^C^	22.4 ± 1.8^B^	26.1 ± 2.4^A^	20.2 ± 1.5^BC^	25.7 ± 1.5^A^
Serum LDL-C	61.7 ± 4.9^B^	68.9 ± 5.7^A^	63.4 ± 4.4^AB^	56.5 ± 5.3^B^	68.6 ± 5.9^A^	57.3 ± 5.3^B^
Serum triglyceride	49.7 ± 4.8^A^	48.3 ± 5.2^A^	48.5 ± 3.8^A^	49.5 ± 5.2^A^	43.2 ± 4.8^B^	42.5 ± 5.1^B^
Liver TG (mg/g tissue)	58.3 ± 5.3^C^	76.5 ± 6.2^A^	74.9 ± 6.2^A^	68.2 ± 6.1^B^	63.3 ± 5.6^C^	62.7 ± 5.2^C^
Liver cholesterol (mg/g tissue)	15.3 ± 1.6^C^	20.4 ± 1.6^A^	19.0 ± 2.0^AB^	17.7 ± 1.2^B^	16.2 ± 1.5^C^	16.4 ± 1.3^C^
Liver glycogen (mg/g tissue)	28.6 ± 2.1^B^	23.1 ± 2.0^C^	24.5 ± 12.9^C^	29.8 ± 2.0^B^	28.5 ± 1.8^B^	34.8 ± 4.2^A^
Serum glucose	99.1 ± 8.3^BC^	114 ± 5.0^A^	108 ± 7.3^AB^	95 ± 4.4^C^	103 ± 5.8^B^	106 ± 6.0^B^
Serum insulin	1.31 ± 0.14^C^	1.75 ± 0.17^A^	1.64 ± 0.16^AB^	1.53 ± 0.14^B^	1.65 ± 0.17^AB^	1.51 ± 0.17^B^
HOMA-IR	6.01 ± 0.65^D^	9.23 ± 0.84^A^	8.2 ± 0.78^B^	6.73 ± 0.53^C^	7.89 ± 0.72^B^	7.42 ± 0.69^BC^
Serum NEFA	0.31 ± 0.09^D^	0.67 ± 0.11^A^	0.59 ± 0.10^AB^	0.48 ± 0.09^BC^	0.53 ± 0.11^B^	0.42 ± 0.09^C^

Values are means ± standard deviation. HDL-C, high density lipoprotein cholesterol; LDL-C, low density lipoprotein; HOMA-IR, homeostatic model assessment for insulin resistance; NEFA, nonesterified fatty acids. Rats consumed high fat diets containing 0.2% (low dosage, -L) and 0.6% (high dosage, -H) of the mixture of mulberry extracts and silk amino acid (MS) and 0.6% cellulose (disease-control) for 12 weeks. MS1:3 and MS1:5 mixture contained 1:3 and 1:5 of mulberry extracts and silk amino acid, respectively. Sham rats were fed the same diet with the disease-control group as the normal-control.

^A,B,C,D^Means on the same row with different letters were significantly different by Tukey test at p < 0.05.

## Data Availability

The data used to support the findings of this study are included within the article.

## Abbreviations

MB: Mulberry water extracts
SA: Silk amino acids
MS1:3-L: Low dosage of mulberry extracts and silk amino acid (1:3, w/w)
MS1:3-H: High dosage of mulberry extracts and silk amino acid (1:3, w/w)
TG: Triglyceride
NAFLD: Nonalcoholic fatty liver disease
NEFA: Nonesterified fatty acid
VLDL: Very low density lipoprotein
ROS: Reactive oxygen species
NASH: Nonalcoholic steatohepatitis
En%: Percent energy
HOMA: Homeostasis model assessment
ALT: Alanine aminotransferase
AST: Aspartate aminotransferase
*γ*-GPT: *γ*-glutamyl transpeptidase
TNF-*α*: TBARS thiobarbituric acid reactive substance
TNF-a: Tumor necrosis factor-*α*
SOD: Superoxide dismutase
GSH: Glutathione
PCR: Polymerase chain reaction
SREBP-1c: Sterol regulatory element-binding protein-1c
ACC: Acetyl CoA carboxylase
FAS: Fatty acid synthase
CPT-1: Carnitine palmitoyl transferase-1
CYP2E1: Cytochrome P450 2E1
IL: Interleukin
H-E: Hematoxylin and eosin
PAS: Periodic acid–Schiff
PcoA: Principal coordinate analysis
AMOVA: Analysis of molecular variance
IPITT: Intraperitoneal insulin tolerance test
AUC: Area under the curve
OGTT: Oral glucose tolerance test
OUTs: Operational taxonomic units.
